# Some Medical Exchanges

**Published:** 1873-06

**Authors:** 


### SOME MEDICAL EXCHANGES.

"American Practitioner," monthly, $3 a year, Louisville, Ky., edited by Professor David W. Yandell and Dr. Theodore Parvin.

"Boston Medical and Surgical Journal," published in monthly and weekly parts for $4 a year, by David Clapp & Son, at 334 Washington Street, Boston, Mass.

"Buffalo Medical and Surgical Journal," monthly, $3 a year, Julius F Minor, M. D., editor.

"Chicago Medical Examiner," monthly, $3 a year, 797 Wabash Avenue.

"Chicago Medical Journal," monthly, $3 a year, Drs. Allen and Hay, editors.

"Dental Cosmos," monthly, $2.50 annually, Philadelphia, Pa., and 767 Broadway, New York.

"Dental Register," monthly, $3 a year, Professor Taft, editor, 167 Walnut Street, Cincinnati, O.

"Druggists' Circular," monthly, $1.50 a year, Dr. L. V. Newton, editor, 36 Beekman Street, New York.

"Eclectic Medical Review," monthly, $2 a year, 166 West 34th Street, New York, Dr. E. S. McLellan, editor.

"Good Health," monthly, $2 a year, published by Alexander Moore, 11 Bromfield Street, Boston, and American News Co., 121 Nassau Street, N. Y.

"Gynæcological Journal," devoted to the advancement of the knowledge of the diseases of women; monthly, $2.50 a year, 18 Tremont St., Boston, Mass.

"Homœopathic American Observer," monthly, $2.50 a year, Detroit, Mich.

"North American Journal of Homœopathy," quarterly, $4 a year, G. Lillienthal, editor, 230 West 25th Street, New York, and Trübner & Co., London.

"Herald of Health," $2 a year, issued monthly by Wood & Holbrook, 15 Laight Street, New York. Hydropathic.

"Journal of Applied Chemistry," monthly, $1.50 a year, by Dexter & Co., 17 Spruce Street, New York.

"Journal of Psychological Medicine," being a quarterly review of Diseases of the Nervous System, Medical Jurisprudence, and Anthropology. Edited by William A. Hammond, M. D., Professor of Diseases of the Mind and Nervous System at Bellevue Hospital; published at $5 a year, by D. Appleton & Co., 551 Broadway, New York, and Trübner & Co., London.

"London Lancet," $5 a year, published monthly, simultaneously with the London edition, by William C. Herald, Esq., agent, 121 Williams Street, New York. Postage 24 cents a year.

"Medical Investigator," monthly, $3 a year, 66 Lake Street, Chicago, Ill.

"Medical and Surgical Reporter" (Dr. Butler's), weekly, $5 a year, 115 South Seventh Street, Philadelphia, and 194 Broadway, New York.

"Medical Times," weekly, $4 a year, published by J. B. Lippincott & Co., 717 Market Street, Philadelphia, Pa.

"Ohio Medical and Surgical Reporter," $1.50 a year, Cleveland, Ohio.

"Phrenological Journal," $3 a year, monthly, published by S. R. Wells, 389 Broadway, New York.

"Science of Health," monthly, $2 a year, by same house.

"Medical Record," semi-monthly, $4 a year, 27 Great John Street, New York.

